# miR-320b, a Future Expected New Biomarker for Type 2 Diabetes Mellitus Induces Dysglycemia by Targeting PTEN

**DOI:** 10.1155/2024/5540062

**Published:** 2024-10-28

**Authors:** Jinxingyi Wang, Ruyu Tao, Hanshuai Hu, Jiejie Gao, Yang Liu, Jie Xia, Xue Lan, Yanan Di

**Affiliations:** Department of Pharmacy, The Second Affiliated Hospital of Guizhou Medical University, Kaili 556000, China

**Keywords:** diabetes mellitus, glucose consumption, miR-320b, PTEN

## Abstract

**Background:** Type 2 diabetes mellitus (T2DM) has emerged as a global epidemic issue, with high rates of disability and fatality. Traditional diagnostic biomarkers are typically detected once a metabolic imbalance has already occurred, thus the development of early diagnostic biomarkers is crucial for T2DM. Metabolomics studies have identified several predictive biomarkers for T2DM, including miR-320. Our previous research found that miR-320b was significantly downregulated in T2DM patients, but the underlying mechanism remains unclear. Therefore, this study was designed to investigate the significance of miR-320b for T2DM diagnosis and to explore the involved molecular mechanism.

**Methods:** A total of 50 patients with T2DM and 80 sex- and age-matched healthy subjects were selected. The plasma miR-320b of all participations was detected by qRT-PCR and its correlations with other biomarkers of T2DM were analyzed. Besides, the expression of miR-320b in HepG2 cells was suppressed by miRNA inhibitors. Then the glucose consumption of HepG2 cells was measured. The target gene of miR-320b was predicted by four bioinformatics tools and intersected these prediction results by Venny method. The T2DM relevant target genes were identified by the GeneCards database. To ensure disease relevance, these T2DM relevant target genes were subsequently intersected with the target genes of miR-320b. Protein–protein analysis (PPI) was used to screening the gene with the most connections in these target genes. Finally, the target gene of miR-320b specific to T2DM was confirmed directly by luciferase reporter assay. The expression of target gene in HepG2 cell culture supernatant and plasma of all participations was detected.

**Results:** Our results showed that the expression level of miR-320b was significantly lower in T2DM patients compared to the healthy controls. It was negatively correlated with fasting plasma glucose (FPG), glycated hemoglobin (HbA1C), and homeostasis model assessment of insulin resistance (HOMA-IR), but positively with HOMA-*β*. The glucose consumption of HepG2 cells in the miR-320b inhibitor group was significantly lower compared to inhibitor-NC and blank control group. We predicted and confirmed that phosphatase and tensin homolog (PTEN) was the direct target gene of miR-320b using Bioinformation tools and luciferase reporter assay. Moreover, the concentration of PTEN was significantly higher in the HepG2 cell culture supernatant and plasma of T2DM patients.

**Conclusions:** Our research demonstrated a negative correlation between miR-320b and FPG, HbA1C, and HOMA-IR, while exhibiting a positive correlation with HOMA-*β*. Suppressing miR-320b expression would impair glucose consumption of HepG2 cells through PI3K pathway by targeting PTEN. These results suggest that miR-320b may be a potential biomarker for diagnosing T2DM and a promising target for therapeutic intervention.

## 1. Introduction

Diabetes mellitus has become a global health challenge, with high disability and fatality rates affecting over 451 million individuals worldwide [1, 2]. This number is predicted to increase to approximately 693 million by 2045 [3]. Type 2 diabetes mellitus (T2DM) accounts for nearly 90%–95% of all cases [4]. T2DM is generally diagnosed using biomarkers such as fasting plasma glucose (FPG), oral glucose tolerance test, and glycated hemoglobin (HbA1C), which is only used after an individual already exhibits metabolic imbalance [5]. Once metabolic syndrome occurs, the symptoms of T2DM can only be delayed, but not completely eliminated. Hence, early diagnosis and development of more effective therapies for T2DM are of paramount importance. Insulin resistance (IR) is a critical contributor to the pathogenesis of T2DM and often begins years before glucose imbalance occurs [6, 7]. Serval factors, such as genetic predisposition and epigenetics [6], have been implicated for IR, but the molecular mechanisms underlying IR remain only partially understood.

MicroRNAs (miRNAs), a class of small noncoding RNAs consisting of 19–25 nucleotides, have been demonstrated to play significant roles in various biological processes and diseases by regulating genes at the post-transcriptional level. An increasing number of researchers have indicated that miRNAs play a critical role in the onset and development of T2DM, particularly in relation to insulin signaling pathways and IR [8]. Several studies have indicated that miRNAs functionally interact with phosphatase and tensin homolog (PTEN) deleted on chromosome 10, a negative regulator of the insulin signaling pathway, and contribute to the progression of T2DM to diabetic nephropathy by aggravating IR [9, 10]. These findings highlight the potential of miRNAs as novel diagnostic biomarkers and therapeutic targets for overcoming IR and T2DM.

In our previous study, we discovered that plasma miR-320b, a member of the human miR-320 family, was significantly lower in patients with T2DM compared to healthy population [11]. The miR-320 family is highly conserved and exists only in vertebrates. Some studies have indicated that miR-320 expression levels were lower in polycystic ovary syndrome women with IR [12]. However, there is limited understanding of the association between miR-320 and T2DM-associated IR, as well as other T2DM diagnostic biomarkers such as FPG and HbA1C. The HepG2 cell line, a human hepatic embryonal tumor cell line that exhibits a hepatocyte-like phenotype and demonstrates insulin sensitivity, has been reported to be IR in vitro [13]. Therefore, our study was designed to analyze the correlation between miR-320 and these biomarkers, and to explore its potential mechanism underlying IR in HepG2 cells, which aims to address this knowledge gap and provide a deeper insight into the involvement of miR-320 in T2DM.

## 2. Materials and Methods

### 2.1. Study Participants

This retrospective study included two groups, a T2DM group with 58 T2DM patients and a healthy control group with 80 healthy individuals. T2DM patients were diagnosed based on WHO criteria [14]. Exclusion criteria included T1DM, renal or liver diseases, acute or chronic inflammatory diseases, or other metabolic-related diseases. The control group comprised age- and sex-matched individuals from healthy check-up population with no family diabetes history and normal FPG. The following data on all participants were collected: sex, age, body mass index (BMI), FPG (mmol/L), HbA1C (%), fasting C-peptide (FCP, ng/mL), insulin (FINS, *μ*U/mL), total cholesterol (TC), and triglyceride (TG). Based on the values of FPC and FINs, the HOMA-IR and *β*-cell function (HOMA-*β*) using the following formulae: HOMA-IR=FPG × FINS/22.5 and HOMA-*β* = FINS × 20/(FPG − 3.5) [15]. We collected fasting plasma samples after clinical biochemistry measurement form all participants and froze the samples at −80°C. The study was approved by the ethics committees of the Second Affiliated Hospital of Guizhou Medical University (Approval NO. #date 20220105). As our samples were collected from the residual plasma of clinical laboratory measurement and all participants were anonymous, as well as there were no adverse effects on the rights and health of subjects, the written informed consent was not obtained.

### 2.2. RNA Extracted and qRT-PCR

Total RNA was extracted using TRIzol reagent (TIANGEN, Beijing, China) according to the manufacturer's instructions. The purity and concentration of the extracted RNA were determined using NanoDrop® ND-200 and agarose gel electrophoresis. The plasma miRNA-320b level was detected using quantitative real-time PCR (qRT-PCR). The RNA was reverse-transcribed using PrimeScript™ RT reagent Kit with gDNA Eraser (TaKaRa, Beijing, China) and assessed using SYBR Green method (TaKaRa, Beijing, China). The relative miR-320b levels were normalized to U6 using the 2^−△△^Ct method. The sequences of the primers for qRT-PCR are presented in Table 1.

### 2.3. Cell Culture and Transfection

HepG2 cells were purchased from the Chinese Academy of Medical Science (Shanghai, China) and cultured with DMEM, which contained 10% fetal bovine serum (FBS) and was supplemented with 100 units/mL of penicillin and 100 *μ*g/mL of streptomycin. The cells were maintained at 37°C with a 5% CO_2_ humid atmosphere. The supernatants were collected and stored at −20°C for the glucose consumption assays. MiR-320b inhibitor, and its corresponding negative controls (inhibitor-NC) were purchased from Guangzhou RiboBio Co., Ltd. Cells in logarithmic growth phase were seeded into a 6-well plate. When the cells reached 80%–90% confluency, the miR-320b inhibitor and inhibitor-NC at a concentration of 50 nmol/L were transected into HepG2 cells using lipofectamine 2000 (Invitrogen, USA), following the manufacturer's instructions. A blank control group (CON) was established for the cells that received no treatment. After 0 h, 12 h, and 24 h, the supernatants were collected for glucose and PTEN concentration determination.

### 2.4. Glucose Consumption Assay

Following the transfection, 100 nm insulin was added to HepG2 and incubated for 15 min. Subsequently, the cells were cultured in DMEM. The levels of glucose in the cell supernatant were determined at 0, 12, and 24 h of transfection using a glucose assay kit (BioSino, Beijing, China) based on the method of glucose oxidase. The glucose concentration at 0 h was considered as baseline level. The value for glucose consumption was calculated as the difference between the glucose concentration at 0 and 12, 24 h. For each experiment, three independent tests were conducted.

### 2.5. Screening of Target Genes of miR-320b Specific to T2DM

The target genes of miR-320b were predicted using four bioinformatic tools: Target Scan, MIRDB, DIANA TOOLS, and miRmap). The Venny method, an online platform for data analysis and visualization, was used to perform an intersection of the target genes of miR-320b predicted by bioinformatic tools [16]. Then the relevant target genes associated with T2DM were identified by the GeneCards database (https://www.genecards.org), utilizing “Type 2 diabetes mellitus” as the search term and restricting the species to “*Homo sapiens*.” To ensure disease relevance, these target genes specific to T2DM were subsequently intersected with the intersection of target genes of miR-320b using Venny method.

The STRING database is employed to search for protein-protein interactions (PPI) expressed by target genes of miR-320b specific to T2DM, and the result of PPI as visualized using the Cytoscape_3.9.0 software [17].

### 2.6. Dual Luciferase Reporter Assay

**Figure 1:**
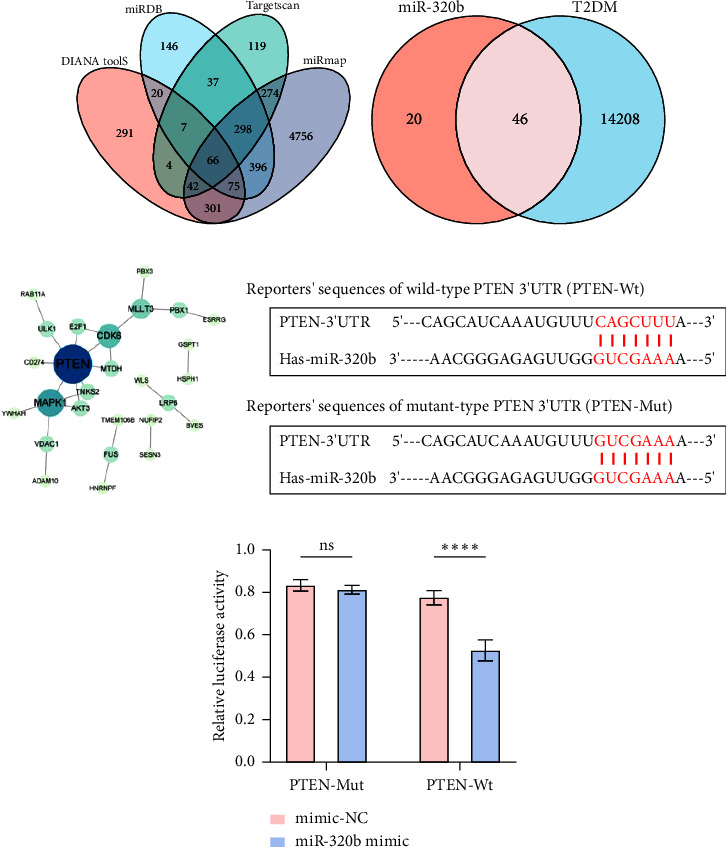
PTEN as a downstream target gene of miR-320b. (a) Venn diagram was constructed with four miRNA databases (pink circle, DIANA ToolS, blue circle miRDB, green circle, targetscan, purple, miRmap) and 66 candidate target genes were obtained by Venn diagram. (b) Forty-six target genes of miR-320b specific to T2DM were obtained by Venn diagram. (c) PPI network showed that PTEN with the highest degree value. The larger the diameter and the deeper color of the circle, the larger the degree value. (d) Putative wild-type (PTEN-Wt) and mutant-type (PTEN-Mut) of miR-320b binding sides (red Letters) in PTEN 3′ UTR sequence of dual-luciferase reporter vector. (e) Dual-luciferase reporter assay of the inhibitory activity of miR-320b significantly upon co-transfection with PTEN-Wt reporter, miR-320b-mimic or mimic-NC, and PTEN-Mut reporter, miR-320b-mimic or mimic-NC in HepG2 cells (⁣^∗∗∗∗^*p* < 0.0001, ns., not significance).

Bioinformatic tool Target Scan was used to predict the binding sides of most likely target gene of miR-320b. The wild-type (PTEN-Wt) and mutant-type (PTEN-Mut) 3′untranslated region (3′UTR) sequence (shown in Figure 1(c)) of PTEN were synthesized into the luciferase vector of Pisicheck-2, respectively. HepG2 cells were seeded into 24-well plates for 24 h before transection. Luciferase constructs with PTEN-Wt or PTEN-Mut sequence, miR-320b mimic or corresponding negative controls (mimic-NC) were co-transfected into HepG2 cells using Lipofectamine 2000. At 48 h following transfection, the cells were lysed using 100 *μ*L lysis solution. The activities of firefly and renilla luciferase were measured using SpectraMax M5. The reporter gene activity was normalized to renilla luciferase activity.

### 2.7. ELISA for PTEN Determination

The measurement of plasma and cell supernatant PTEN concentration was performed using an ELISA kit procured from Abcam (Cambridge, MA, USA, Cat. No. ab206979). A standard curve was established by plotting the subtracted absorbance value for each standard concentration (*Y*-axis) against the target protein concentration (*X*-axis) of the standard.

### 2.8. Statistical Analysis

A post-hoc power calculation was performed using GPower 3.1 and result showed that we had an adequate sample size, which could achieve power of 80.3% at a two-sided *α* level of 0.05 and effect size 0.5. Statistical analysis was performed using GraphPad Prism Version 9.5.1 software. All data were shown as mean ± SD and *p* < 0.05 was considered to be statistically significant. Unpaired *t*-test was used to compare parameters between T2DM patients and controls for data that was normally distributed data, while Mann–Whitney test for non-normally distributed data. Spearman correlation analysis was employed to determine the association between miR-320b and other biomarkers, while One-way ANOVA was used to analyze comparisons among multiple groups.

## 3. Results

### 3.1. Clinical and Biochemical Characteristics of the Study Participants

The clinical and biochemical characteristics of the study participants are presented in Table 2. There was no statistically significant difference of the male/female ratio (56.67 vs. 51.25%) and age 50.98 ± 8.038 versus 47.60 ± 12.73) in T2DM group and healthy control group. The mean ± SD of FPG, glycosylated hemoglobin (HbA1C) was significantly higher in T2DM group compared to that's in healthy control group, with values of (12.60 ± 5.009 vs. 5.217 ± 0.4664), (10.42 ± 3.171 vs. 5.413 ± 0.416), respectively. Conversely, the mean ± SD of (FINS), FCP were significantly lower in T2DM group compared to that's in healthy control group, with values of (9.435 ± 2.813 vs. 11.480 ± 3.8440), (2.054 ± 1.228 vs. 2.540 ± 0.7792), respectively. These results revealed the comparability of research subjects between T2DM group and healthy control group.

### 3.2. miR-320b Expression Level Was Significantly Lower in Patients With T2DM

**Figure 2:**
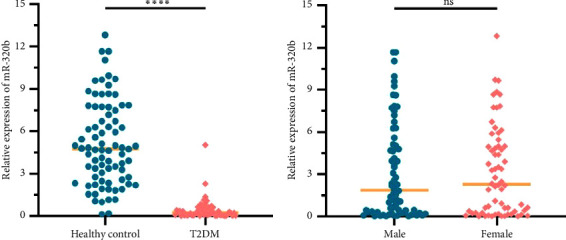
Expression of miR-320b by qRT-PCR analysis in plasma samples from different populations. (a) Expression of miR-320b in T2DM patients and healthy control individuals (⁣^∗∗∗∗^*p* < 0.0001). (b) Expression of miR-320b in male and female (ns., not significant). Data are represented by scatter diagram. The *p* values were calculated by unpaired *t*-test.

In order to explore the distribution of miR-320b expression level in different subjects, we compared the plasma miR-320b expression levels between patients with T2DM and healthy controls by unpaired *t*-test. The results demonstrated that the miR-320b expression level was significantly lower in T2DM patients compared to the healthy controls with mean ± SD of 5.279 ± 3.206 and 0.489 ± 0.774, respectively (Figure 2(a)). Additionally, there was no significant difference in the expression of miR-320b between male and female, with mean ± SD of 3.190 ± 3.384 and 3.342 ± 3.542, respectively (Figure 2(b)).

### 3.3. The Correlation of Plasma miR-320b Expression Levels With Traditional Biomarkers of T2DM

**Figure 3:**
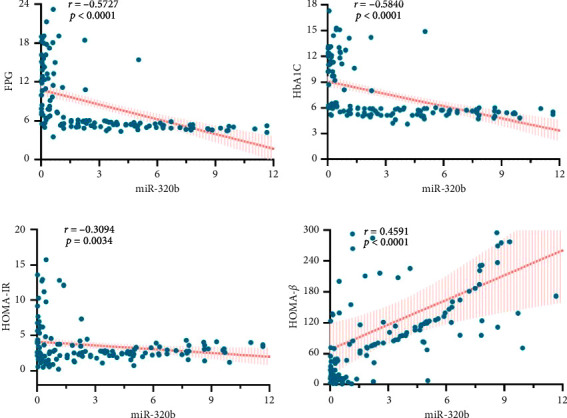
Correlation of miR-320b with the concentration of traditional diagnostic biomarkers in all study individuals. (a–d) The expression of miR-320b was significantly correlated with FPG (a), HbA1C (b), HOMA-IR (c), and HOMA-*β* (d). Correlation coefficient *r* and its significance were calculated by Spearman correlation analysis.

Since there was significant difference of miR-320b expression in T2DM and healthy populations, we speculate whether it can be used as a potential biomarker for T2DM diagnosis. Therefore, we analyzed the correlation of plasma miR-320b expression levels with traditional biomarkers of T2DM using Spearman's correlation analysis. The results indicated that miR-320b was negatively correlated with FPG (*r* = −0.5727, *p* < 0.0001, Figure 3(a)), HbA1C (*r* = −0.5840, *p* < 0.0001, Figures 3(a) and 3(b)), and HOMA-IR (*r* = −0.3094, *p*=0.0034, Figure 3(c)), but positively correlated with HOMA-*β* (*r* = −0.4591, *p* < 0.0001, Figure 3(d)).

### 3.4. Glucose Consumption Was Impaired by Suppression of miR-320b

**Figure 4:**
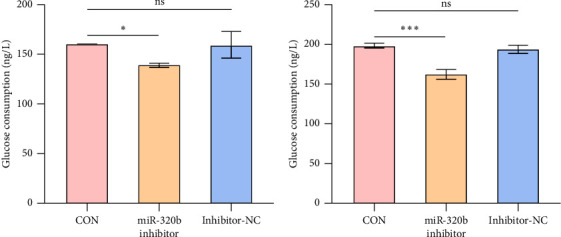
Suppression of miR-320b expression impaired glucose consumption. (a) Glucose consumption in miR-320b inhibitor group, inhibitor-NC group and CON group after 12 h post-transfection (⁣^∗^*p* < 0.05, ns., not significance). (b) Glucose consumption in miR-320b inhibitor group, inhibitor-NC group and CON group after 24 h post-transfection (⁣^∗∗∗^*p* < 0.001, ns., not significance).

To explore whether miR-320b is the causal contributor to T2DM, we used a miR-320b inhibitor to suppress the expression of miR-320b in HepG2 cells and assessed its influence on glucose consumption after 12 and 24 h post-transfection. The results showed that the glucose consumption in the miR-320b inhibitor group was significantly lower compared to inhibitor-NC and blank control group (CON) at 12 h (Figure 4(a)) and 24 h post-transfection (Figure 4(b)), whereas there were no differences between the inhibitor-NC and group.

### 3.5. PTEN as a Downstream Target Gene of miR-320b

To further explore the molecular mechanism of miR-320b in T2DM, we took advantage of multiple bioinformatic databases for target gene prediction. The four online databases predicted 847, 1045, 806, and 6208 target genes, respectively. By overlapping with the Venn Map, a total of 66 candidate target genes were gained (Figure 1(a)). A total of 14,254 T2DM-related target genes were obtained from the GeneCards database. Subsequently, 66 miR-320 target genes were intersected with the 14,254 T2DM-related target genes, resulting in 46 common target genes as target genes of miR-320b specific to T2DM (Figure 1(b)). The PPI expressed by these 46 target genes were predicted and visualized using STRING database and Cytoscape_3.9.0. software. The result showed that the node with the highest degree was PTEN, indicating the PTEN was the most possible target genes of miR-320b specific to T2DM (Figure 1(c)).

To assess whether miR-320b directly targets PTEN, the binding site for miR-320b in the 3′UTR region of PTEN was predicted using TargetScan. Then the luciferase-based reporters constructed either with PTEN-Wt or PTEN-Mut sequence (Figure 1(d)) were synthesized. The dual-luciferase report assay was performed and results shown that the miR-320 mimic significantly suppressed the luciferase activity of PTEN-Wt compared to the mimic-NC group (*p* < 0.0001), while luciferase activity was rescued by mutation of their binding region (Figure 1(e)).

### 3.6. T2DM Patients and miR-320b Inhibitor Treated HepG2 Cells With Higher Expression Levels of PTEN

**Figure 5:**
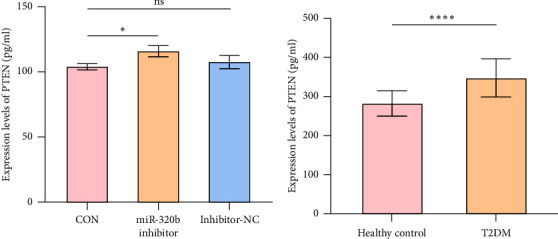
T2DM patients and miR-320b inhibitor treated HepG2 cells with higher expression levels of PTEN (a) the expression levels of PTEN in HepG2 cells supernatant of miR-320b inhibitor group, inhibitor-NC group and CON group (⁣^∗^*p* < 0.05, ns., not significant). (b) The expression levels of PTEN in T2DM patients and healthy controls (⁣^∗∗∗^*p* < 0.001, ns., not significant).

To explore whether miR-320b affects the PTEN expression, we transfected miR-320b inhibitor and inhibitor-NC into HepG2 cell and measured PTEN protein expression levels in cell culture supernatant using ELISA at 24 h post-transfection. Our results revealed that the PTEN protein expression level of miR-320b in miR-320b inhibitor group was significantly higher compared to CON group (*p* < 0.05), while there was no significantly difference between miR-320b inhibitor-NC group and CON group (Figure 5(a)). Additionally, we measured the concentration of plasma PTEN in patients with T2DM and healthy controls. The results showed that the concentration of plasma PTEN was significantly higher in T2DM group compared to the healthy controls (*p* < 0.01) (Figure 5(b)).

## 4. Discussion

The miR-320 family consists of 5 members: miR-320a, miR-320b, miR-320c, miR-320d, and miR-320e. Several studies have demonstrated that miR-320 contributed to glucose and lipid metabolism by different signaling pathways [18]. In our study, we assessed the expression of miR-320b in both T2DM patients and healthy controls. Our results showed that the expression of miR-320b was significantly lower in T2DM patients, consistent with our previous study [11]. Similarly, a prospective study showed lower plasma levels of miR-320 in both prediabetes and T2DM [19]. However, it is worth mentioning that the expression of this miR-320 family is still a controversial issue. Another study observed an upregulation of circulating miR-320 in T2DM patients [20]. These controversies may be attributed to differences in sample size or study populations as well as different members of the miR-320 family.

Additionally, we observed correlations between the expression levels of miR-320b and traditional biomarkers of T2DM. We found a negative correlation between the expression level of miR-320b and FPG, HbA1C. Furthermore, miR-320b exhibited a negative correlation with HOMA-IR but a positive correlation with HOMA-*β*. It is worth noting that HOMA-IR and HOMA-*β* are critical parameters for IR and pancreatic *β* cell function, both of which play crucial roles in the pathogenesis of T2DM. This suggests that changes in miR-320b may occur even in the absence of elevated glucose levels. While some miRNAs have been reported to be correlated with individual biomarkers such as HbA1C, HOMA-IR, or FPG (e.g., miR-135 [21], miR-18a [22], and miR29a [23]), those simultaneously correlated with multiple biomarkers of T2DM are rare. Our results indicated that not only does miR-320b reflect the levels of FPG and HbA1C, but also correlates with HOMA-IR and HOMA-*β*. Therefore, it is reasonable to speculate that miR-320b could be potentially served as a new early biomarker for T2DM.

This study is a cross-sectional investigation. Thus, it remains unclear whether miR-320b serves as a causal factor in the development of T2DM. By employing a miR-320b inhibitor to suppress the miR-320b expression, we observed its effect on glucose consumption in HepG2 cells. The results demonstrated that when miR-320b expression was suppressed, glucose consumption in HepG2 cells was significantly reduced, indicating that inhibiting miR-320b expression may impair glucose uptake and ultimately result in hyperglycemia.

To provide further evidence of the mechanism through which miR-320b regulates glucose consumption, and for avoiding the false targets, we identified its target genes by four bioinformatic tools and intersected the prediction results. To ensure disease relevance, we identified the relevant target genes associated with T2DM by the GeneCards database and then intersected these genes to target genes of miR-320b. Finally, a total of 46 target genes were obtained as the target genes of miR-320b specific to T2DM. For searching the genes with the most connections in these 46 target genes, the PPI network was instructed and found protein PTEN with highest degree, indicating the PTEN was the most possible target genes of miR-320b specific to T2DM. And then, the luciferase reporter assay directly confirmed that PTEN was the target gene of miR-320b.

Additionally, we observed that miR-320b inhibitor significantly increased PTEN protein expression in HepG2 cells supernatant. Similarly, T2DM patients exhibited higher plasma levels of PTEN protein and lower levels of miR-320b. Although PTEN was first defined as a tumor suppressor [24], emerging evidence suggests that PTEN may also function as a negative regulator of the phosphatidylinositol-3-kinase (PI3K)/AKT pathway, which plays a critical role in glucose metabolism regulation [25]. Insulin binding to the insulin receptor activates PI3K, which then phosphorylate membrane-bond phosphatidylinositol 4,5-bisphosphate (PIP2) to phosphatidylinositol 3,4,5-triphosphate (PIP3). Subsequently, PIP3 activates downstream proteins, including AKT/PKB and then phosphorylate more downstream targets that initiate the anabolic functions of insulin [26, 27]. Therefore, the repression of this pathway is an important mechanism of IR. PTEN, as a negative regulator of this pathway, can dephosphorylate PIP3 back to PIP2 and inhibit the effects of PI3K signaling in response to insulin [28, 29]. As a result, loss-of-function of PTEN can lead to enhanced insulin signaling [30] and protection against IR [31]. Studies have demonstrated that PTEN haploinsufficiency is associated with increased sensitivity [32] and show decreased baseline insulin and glucose levels [33]. Above this, we speculated that the reduction in the levels of miR-320b may lead to an increase in the expression of PTEN protein, which in turn inhibits the PI3K signaling pathway, ultimately resulting in IR and T2DM.

However, there are some limitations in this study that need to be addressed in future research. Firstly, as a single-center, retrospective study with a limited sample size, our findings may not be applicable to other populations. Furthermore, our study did not encompass prediabetic patients. Therefore, it is imperative to conduct a prospective, multicenter study with a larger sample size to further verify the role of miR-320b as an early diagnostic biomarker for T2DM. Secondly, while we hypothesized the mechanisms of miR-320b in T2DM at the cellular level, it is crucial to explore its biological function and specific genetic networkers in animal models in the future studies.

In conclusion, our study has revealed a negative correlation between miR-320b and FPG, HbA1C, HOMA-IR, while demonstrating a positive correlation with HOMA-*β*. Importantly, we have validated that PTEN, an essential inhibitor of the insulin pathway PI3K, is directly targeted by miR-320b and exhibits significantly higher expression among T2DM patients. These findings provide potential diagnostic biomarkers and therapeutic targets that may aid in accessing the IR and pancreatic-*β* cell function of T2DM, ultimately leading to improved treatment for T2DM.

## Figures and Tables

**Table 1:** Sequences of primers used for qRT-PCR.

Gene		Primer sequence (5′-3′)
miR-320b	Forward	AAAAGCTGGGTTGAGAGGGCAA
	Reverse	GGCCAACCGCGAGAAGATG

U6	Forward	CTGCGCAAGGATGACACGCAAATT
	Reverse	GGCCAACCGCGAGAAGATG

**Table 2:** Clinical and biochemical characteristics of T2DM and healthy control participants.

Characteristics	T2DM (*n* = 58)	Control (*n* = 30)	*p* value
Male (%)	53.45%	51.25%	0.6716
Age (years)	47.60 ± 12.73	50.98 ± 8.038	0.0590
FPG (mmol/L)	12.60 ± 5.009	5.217 ± 0.4664	< 0.0001
HbA1C (%)	10.42 ± 3.171	5.413 ± 0.416	< 0.0001
FINS (*μ*U/mL)	9.435 ± 2.813	11.480 ± 3.8440	0.0665
FCP (ng/mL)	2.054 ± 1.228	2.540 ± 0.7792	0.0053

## Data Availability

All data generated or analyzed during this study are included in this published article.
